# An immuno-wall microdevice exhibits rapid and sensitive detection of IDH1-R132H mutation specific to grade II and III gliomas

**DOI:** 10.1080/14686996.2016.1227222

**Published:** 2016-10-04

**Authors:** Akane Yamamichi, Toshihiro Kasama, Fumiharu Ohka, Hiromichi Suzuki, Akira Kato, Kazuya Motomura, Masaki Hirano, Melissa Ranjit, Lushun Chalise, Michihiro Kurimoto, Goro Kondo, Kosuke Aoki, Noritada Kaji, Manabu Tokeshi, Toshio Matsubara, Takeshi Senga, Mika K. Kaneko, Hidenori Suzuki, Masahito Hara, Toshihiko Wakabayashi, Yoshinobu Baba, Yukinari Kato, Atsushi Natsume

**Affiliations:** ^a^ Department of Neurosurgery, Nagoya University Graduate School of Medicine, Nagoya, Japan; ^b^ Department of Applied Chemistry, Graduate School of Engineering, Nagoya University, Nagoya, Japan; ^c^ Division of Applied Chemistry, Hokkaido University, Sapporo, Japan; ^d^ Department of Neurosurgery, Mie University Graduate School of Medicine, Tsu, Japan; ^e^ Division of Cancer Biology, Nagoya University Graduate School of Medicine, Nagoya, Japan; ^f^ Department of Regional Innovation, Tohoku University Graduate School of Medicine, Sendai , Japan

**Keywords:** Glioma, isocitrate dehydrogenase 1 mutation, immuno-wall microdevice, rapid diagnosis, precision medicine, 30 Bio-inspired and biomedical materials, 404 Materials informatics / Genomics

## Abstract

World Health Organization grade II and III gliomas most frequently occur in the central nervous system (CNS) in adults. Gliomas are not circumscribed; tumor edges are irregular and consist of tumor cells, normal brain tissue, and hyperplastic reactive glial cells. Therefore, the tumors are not fully resectable, resulting in recurrence, malignant progression, and eventual death. Approximately 69–80% of grade II and III gliomas harbor mutations in the isocitrate dehydrogenase 1 gene (*IDH1*), of which 83–90% are found to be the *IDH1-R132H* mutation. Detection of the *IDH1-R132H* mutation should help in the differential diagnosis of grade II and III gliomas from other types of CNS tumors and help determine the boundary between the tumor and normal brain tissue. In this study, we established a highly sensitive antibody-based device, referred to as the immuno-wall, to detect the *IDH1-R132H* mutation in gliomas. The immuno-wall causes an immunoreaction in microchannels fabricated using a photo-polymerizing polymer. This microdevice enables the analysis of the *IDH1* status with a small sample within 15 min with substantially high sensitivity. Our results suggested that 10% content of the *IDH1-R132H* mutation in a sample of 0.33 μl volume, with 500 ng protein, or from 500 cells is theoretically sufficient for the analysis. The immuno-wall device will enable the rapid and highly sensitive detection of the *IDH1-R132H* mutation in routine clinical practice.

## Graphical abstract

**Figure:**
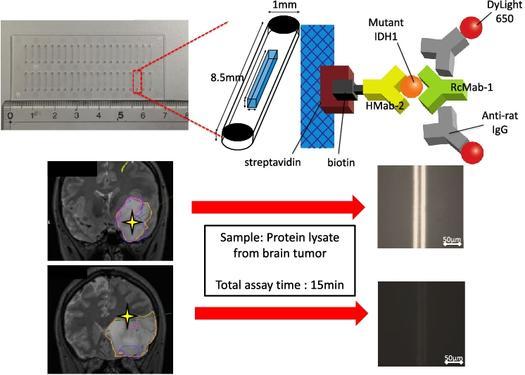

## 1. Introduction

Glioma accounts for approximately 30% of all of central nervous system (CNS) tumors in adults.[1] Gliomas are divided into World Health Organization (WHO) grades I–IV[2]; grade IV glioma, namely glioblastoma (GBM), is the most malignant subtype with a median survival time of approximately 15 months despite extensive surgical resection and chemo-radiotherapy.[3,4] Although grade II and III gliomas (lower grade gliomas, LGGs) are less aggressive than GBM, because of their infiltrative nature, complete surgical removal cannot be achieved. Pathologically, the surrounding marginal tissue is a complex mixture of normal white and gray matters, infiltrative tumor cells, and hyperplastic reactive glial cells. Moreover, in most cases, LGGs appear similar to the normal tissue without clear boundaries between the two. Such non-circumscribed tumors are not fully resectable, resulting in recurrence, malignant progression, and eventual fatality.[5]

Recent comprehensive genetic studies in large cohorts of LGGs revealed that the isocitrate dehydrogenase 1 (*IDH1*) mutation was found in 69–80% of these gliomas.[6–^[7]^ 8] The most significant of the *IDH1* mutations in LGGs substitutes the amino acid residue 132 from arginine to histidine (R132H), accounting for 83–90% of *IDH1* mutations.[9 ^[10]^–11] While the wild-type IDH1 catalyzes the oxidative decarboxylation of isocitrate and produces alpha-ketoglutarate (alpha-KG) in the tricarboxylic acid cycle,[12] the mutant IDH1 converts alpha-KG further into 2-hydroxyglutarate, which, as an oncogenic metabolite, plays several crucial roles in the initiation of glioma.[13] More importantly, the *IDH1* mutation is rarely found in other CNS tumors,[14] and regardless of their locations (i.e. in the tumor core or in the margin), and every tumor cell in an LGG harboring the mutation of *IDH1* expresses the mutated IDH1.[6] These facts suggest that detection of the *IDH1* mutation would enable clinicians to distinguish LGGs from other CNS tumors and to better delineate the ambiguous tumor margin from the normal brain. The *IDH1* mutation is a potential biomarker; however, the only means of detecting this mutation in routine clinical practice thus far are direct sequencing [15] and immunohistochemistry with anti-IDHR132H antibody,[16] both of which are time-consuming and labor-intensive.

We previously constructed immuno-wall devices to enable rapid molecular analysis (manuscript in preparation). These immuno-wall structures were fabricated with a photo-polymerizing polymer placed inside of microchannels on a plastic chip. This device enables the analysis of molecular characteristics in under 15 min using only a small sample.

In this study, we developed a novel immuno-wall device to detect the *IDH1-R132H* mutation in glioma. We found high sensitivity for *IDH1-R132H* even in small amounts of tumor tissue.

## 2. Methodology

### 2.1. Ethics statement 

This study was approved by the institutional review board at Nagoya University Hospital and complied with all provisions of the Declaration of Helsinki. Informed consent was obtained before the operation from all the patients. 

### 2.2. Cell lines 

U87 and immortalized normal human astrocytoma (NHA), expressing either mutated IDH1 (U87-IDH1-R132H, NHA-IDH1-R132H, respectively) or wild-type IDH1 (U87-wtIDH1, NHA-wtIDH1, respectively) were kindly donated by Dr Russell O. Pieper of the University of California, San Francisco, CA, USA. These cell lines were maintained in Dulbecco’s modified Eagle’s medium (DMEM; Sigma-Aldrich, St Louis, MO, USA), containing 10% heat-inactivated fetal bovine serum (FBS; Thermo Fisher Scientific Inc., Waltham, MA, USA), 100 units ml^–1^ of penicillin and 100 μg ml^–1^ of streptomycin (Thermo Fisher Scientific Inc.) at 37°C in a humidified atmosphere of 5% CO_2_. 

### 2.3. Intra-operative collection of tumor tissues 

Fresh tumor samples, 5–10 mm in diameter, were collected intraoperatively from 10 patients whose tumors were resected at Nagoya University Hospital in 2015. The location of each sample was recorded stereotactically in an intraoperative navigation system (Brainlab, Munich, Germany). Each tumor tissue was dissected into three pieces for the immuno-wall assay, immunohistochemistry, and DNA sequencing.

### 2.4. Preparation of protein lysate 

Cell pellets were mechanically broken down in RIPA buffer (Wako, Osaka, Japan), which contained protease inhibitor (Wako), and centrifuged at 15,000 rpm for 5 min at 4 °C. Supernatants were collected and analyzed with the immuno-wall assay. In order to lyse the tumor tissues, the tissues were placed in 1.5 ml tubes containing 200 μl RIPA buffer, a protease inhibitor, and resin beads, which were then collectively ground using pestles from a sample-grinding kit (GE Healthcare, Little Chalfont, UK). The lysate was then centrifuged at 15,000 rpm for 5 min at 4 °C and the supernatants were collected and analyzed. Approximately 100 μg of protein was extracted from 10^5^ cells. 

### 2.5. Western blot analysis

Cell lysates were boiled at 100 °C for 5 min in SDS sample buffer (New England Biolabs, Ipswich, MA, USA) containing 42 mM dithiothreitol (Cell Signaling Technologies, Danvers, MA, USA). Next, the samples (50 μg) were applied to each well and resolved on a 10% polyacrylamide gel (Bio-rad, Hercules, CA, USA). Proteins were transferred to a polyvinylidene difluoride membrane (GE Healthcare), blocked with 5% skim milk in phosphate-buffered saline (PBS) containing 0.1% Tween-20 for 1 h at room temperature, and incubated with HMab-2, RcMab-1, and anti-β-actin (Sigma-Aldrich) for 60 min at room temperature. The membrane was then washed, incubated with horseradish peroxidase-labeled secondary antibodies for 30 min at room temperature, and visualized using an enhanced chemiluminescence method.

### 2.6. Immuno-wall assay 

**Figure 1.**
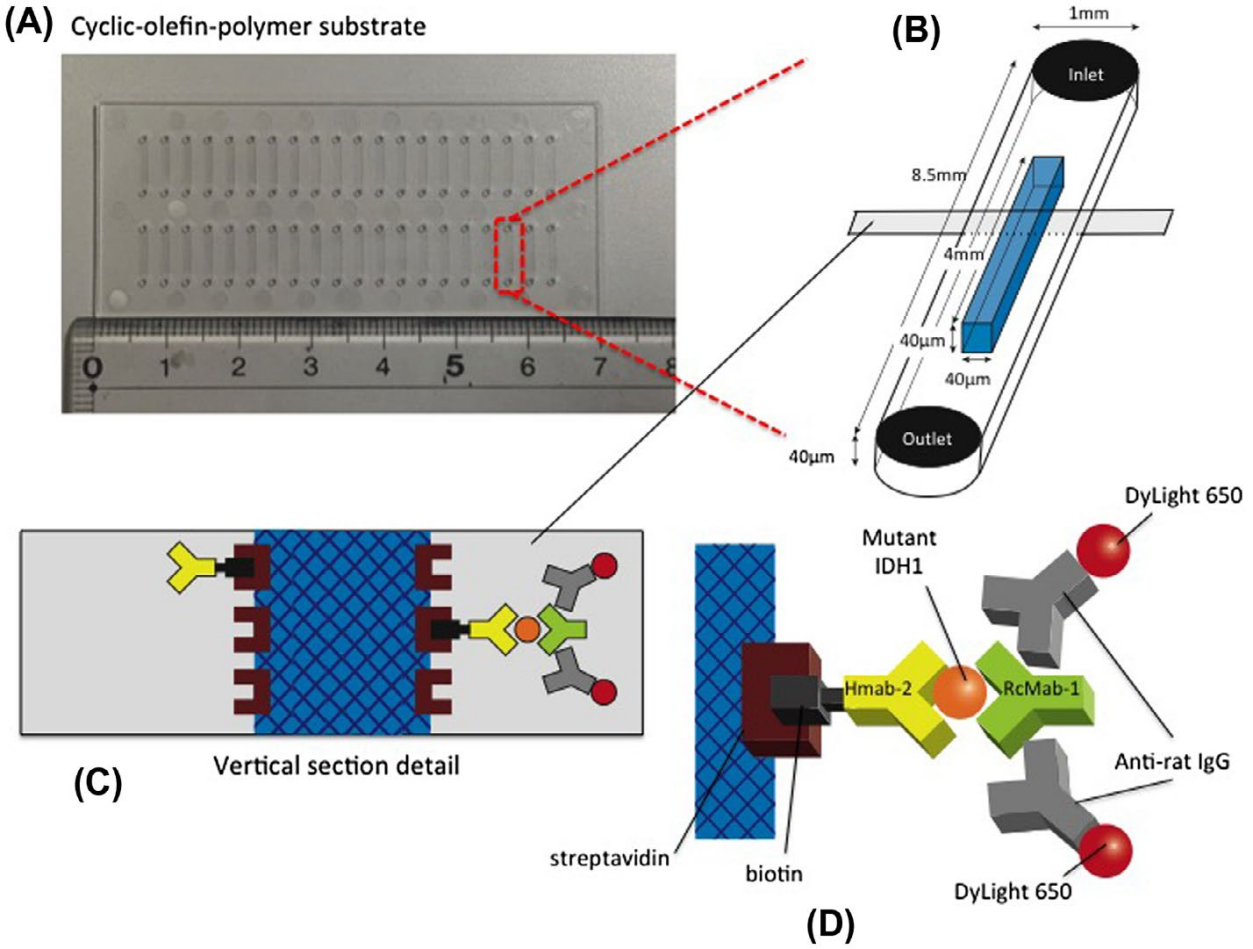
Schematic representation of the immuno-wall device. (A) Immuno-wall chips with 40 microchannels (each 1 mm in width, 40 μm in height and 8.5 mm in length) in a cyclic olefin polymer substrate were constructed using photolithography. (B) The channels were filled with 6% azide-unit pendant water-soluble photopolymer (AWP) and 10 mg ml^–1^ streptavidin. UV light (313 nm, 20 mW cm^–2^) through slits in a photomask was used to immobilize the photoreactive polymer in the center of the channels, before the uncured polymer was washed with PBS. (C, D) A biotinylated anti-R132H-IDH1 antibody (HMab-2), an anti-wild-type IDH1 antibody (RcMab-1), and a fluorescent DyLight650-conjugated goat anti-rat IgG antibody were used to label IDH1-R132H. Note that RcMab-1 is interacted with multiple numbers of DyLight 650-conjugated anti-rat IgGs.

The structure of the immuno-wall device is shown in Figure 1. Immuno-wall chips with 40 microchannels (1 mm width, 40 μm height and 8.5 mm length each) in a cyclic-olefin-polymer substrate were constructed using photolithography. The channel was filled with 6% azide-unit pendant water-soluble photopolymer (AWP; Toyo Gosei, Tokyo, Japan) and 10 mg ml^–1^ streptavidin (Prospec, East Brunswick, NJ, USA). Through slits in a photomask, UV light (313 nm, 20 mW cm^–2^) immobilized the photoreactive polymer in the center of channels, before uncured polymer was removed by washing with PBS. 

A biotinylated anti-R132H mutated IDH1 antibody (HMab-2),[17] an anti-wild-type IDH1 antibody (RcMab-1),[18] and a fluorescent DyLight650-conjugated goat anti-rat IgG antibody (Abcam, Cambridge, UK) were used to label R132H mutated IDH1. PBS, containing 1% bovine serum albumin (BSA; Roche, Basel, Switzerland), was used to dilute the antibodies. PBS, containing 0.5% Tween-20 and 0.5% BSA, was used as a washing buffer.

Biotinylated HMab-2 (1 μl, 50 μg ml^–1^) was injected into inlets, and incubated for 1 h. Microchannels were washed five times with washing buffer. The sample lysate (1.5 μl) was then injected into the inlet and incubated for 5 min followed by incubation of RcMab-1 (1 μl, 50 μg ml^–1^) for 30 s. DyLight650-conjugated goat anti-rat IgG was applied. Finally, the fluorescence was measured and quantified using a fluorescence microscope with a CCD camera and ImageJ software (National Institute of Health, Bethesda, MD, USA).

### 2.7. Direct sequencing and pyrosequencing for IDH1 mutation

DNA was prepared using the QIAmp DNA Mini kit (Qiagen, Hilden, Germany) according to the manufacturer’s instructions. The amount of DNA obtained from the tumor was sufficient for the subsequent genomic analyses. For *IDH1* sequencing, a 129-bp fragment, spanning the sequence encoding the catalytic domain of *IDH1*, including codon 132, was amplified. We applied conventional polymerase chain reaction (PCR) for 35 cycles with denaturation at 95 °C for 30 s, annealing at 56 °C for 40 s, and extension at 72 °C for 50 s, followed by 72 °C for 7 min to complete the extension. Primer sequences were as follows: forward primer: CGGTCTTCAGAGAAGCCATT: reverse primer: GCAAAATCACATTATTGCCAAC. Direct sequencing was performed using the BigDye Terminator v1.1 Cycle Sequencing Kit (Applied Biosystems, Foster City, CA, USA). The reactions were carried out using an ABI 3100 Genetic Analyzer (Applied Biosystems). For pyrosequencing, we applied PCR at 50 cycles with denaturation at 95 °C for 30 s, annealing at 58 °C for 30 s, and extension at 72 °C for 45 s, followed by 72 °C for 2 min to complete the extension. Primer sequences were as follows: forward primer: GGCTTGTGAGTGGATGGGTA: reverse primer: GGGACACCGCTGATCGTTTATGTGTTGAGATGGACGCCTA and universal primer (5′-biotin) GGGACACCGCTGATCGTTTA. Detection and calculation of the frequency of the mutant allele was performed using pyrosequencing technology (Pyrosequencing AB, Uppsala, Sweden) with the sequencing primer: TGGATGGGTAAAACCTATCATCA, according to the manufacturer’s instructions.[19]

### 2.8. Immunohistochemistry

HMab-2 antibody was used for immunohistochemistry. The tumor samples were fixed with 10% formalin and embedded with paraffin. Sections (5-μm thick) were prepared using a microtome (RM2125RT, Leica, Wetzlar, Germany). After deparaffinization and hydration, the sections were incubated in retrieval solution, Tris-EDTA buffer pH 9.0, for 30 min at 100 °C in an electric pot and then blocked with 1.5% normal goat serum (Vector Laboratories, Burlingame, CA, USA) in PBS containing 0.05% Tween-20, at room temperature for 1 h, and were incubated with HMab-2, diluted to 1 μg ml^–1^, overnight at 4 °C. The second labeled polymer from the EnVision HRP kit (Dako; Agilent Technologies, Inc., Santa Clara, CA, USA) was applied and the sections were incubated for 30 min. The substrate-chromogen solution from the DAB Substrate Kit (Vector Laboratories) was applied for 10 min. After washing, the sections were counterstained with hematoxylin and mounted in multi-Mount (Matsunami Glass Ind., Kishiwada, Japan).

### 2.9. Statistical analysis

The statistical significance of the differences between two cell line groups was determined using Student’s *t*-test for the mean fluorescence intensity. *P*-values < 0.05 were considered significant.

## 3. Results 

### 3.1. Sensitivity and specificity of immuno-wall assays

On the basis of our previous study which detected a mutation in lung cancer (manuscript in preparation), we found that 1.5 μl of cell lysate at a concentration of 1.0 mg ml^–1^ was sufficient for conducting an immuno-wall assay. The cell lysate extracted from U87-IDH1-R132H, at a protein concentration of 0.5–2.5 mg ml^–1^, yielded a positive fluorescence intensity along the immuno-wall (Figure S1). Thus, our device detected IDH1-R132H in as little as 0.5 mg ml^–1^ of protein lysates.

**Figure 2.**
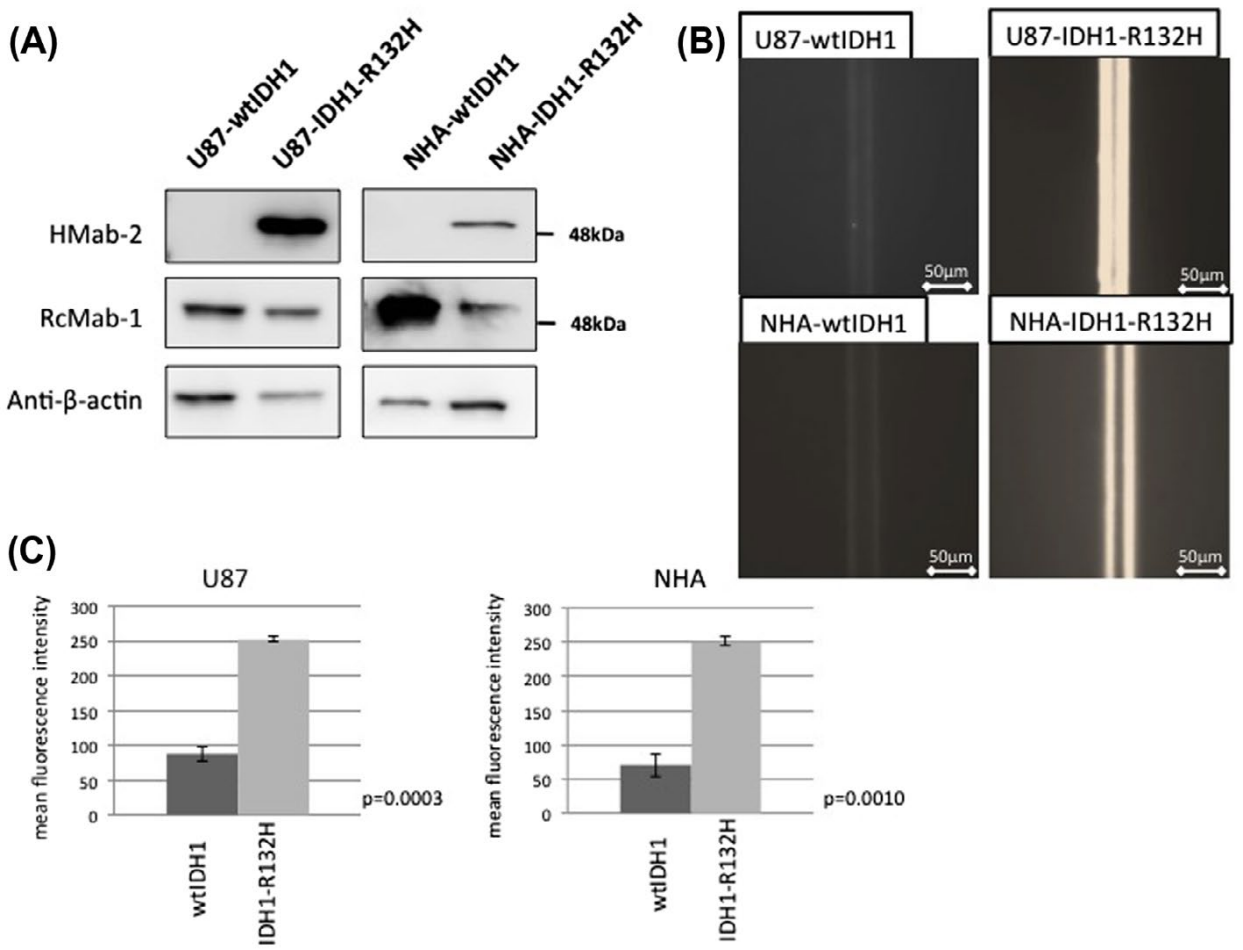
Sensitivity and specificity of the immuno-wall assays. (A) Western blotting confirmed that the anti-IDH1-R132H antibody, HMab-2, was specific to the lysate from NHA-IDH1-R132H and U87-IDH1-R132H cells, whereas the anti-IDH1 antibody, RcMab-1, recognized both wild-type IDH1 and IDH1-R132H in NHA and U87 cells. (B) U87 and NHA cells expressing wild-type IDH1 did not show fluorescence, while both types of cells expressing IDH1-R132H displayed strong fluorescence.

Western blotting confirmed that the anti-IDH1-R132H antibody, HMab-2, was specific to the lysate from NHA-IDH1-R132H and U87-IDH1-R132H cells, whereas the anti-IDH1 antibody, RcMab-1, recognized both wild-type and mutant IDH1 in NHA and U87 cells (Figure 2(A)). In the immuno-wall assay, at the highest protein concentration (3.0 mg ml^–1^), U87 and NHA cells expressing wild-type IDH1 did not show fluorescence while both types of cells expressing IDH1-R132H displayed strong fluorescence. The mean fluorescence intensity was significantly different between cells expressing wild-type IDH1 and IDH1-R132H (Figure 2(B)).

**Figure 3.**
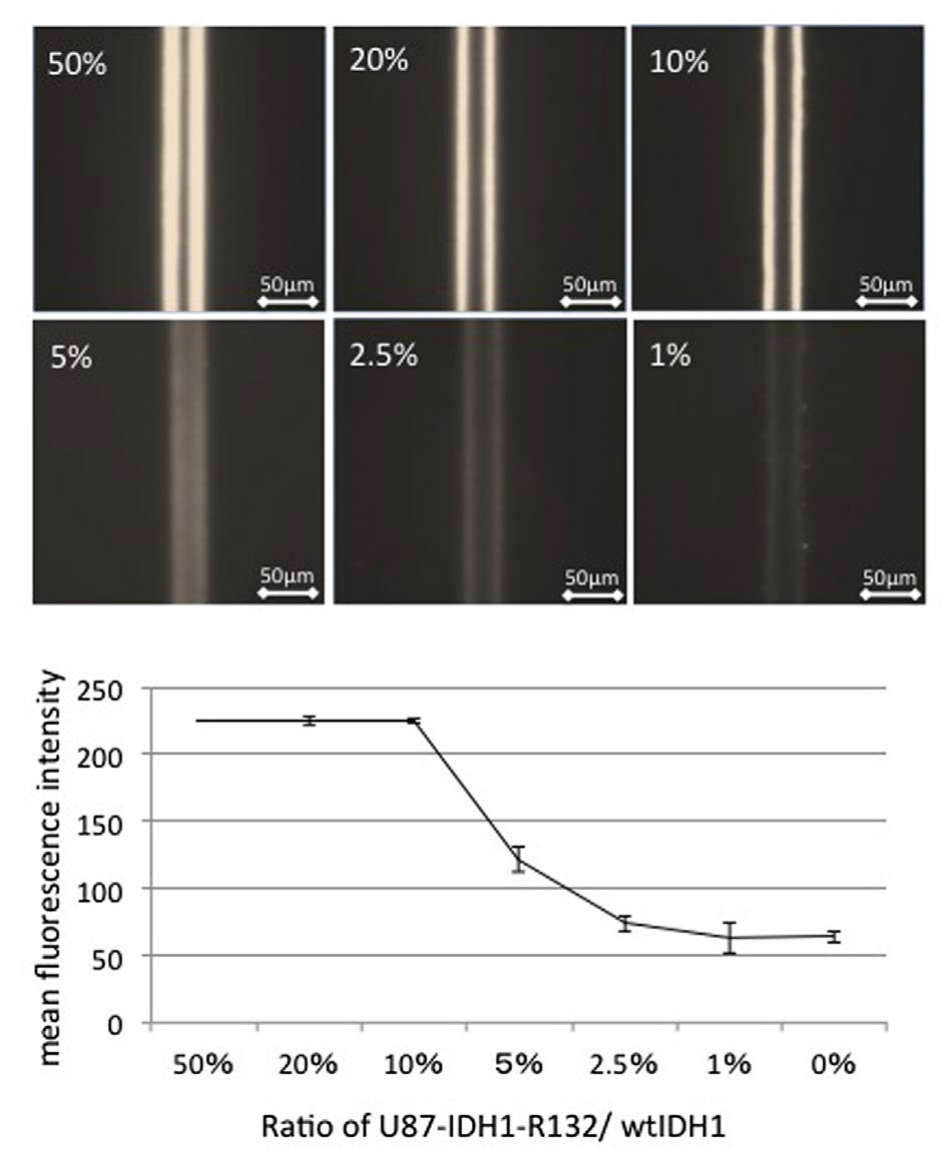
Effect of mutated IDH1/wild-type IDH1 ratio on the detection of mutated IDH1. When mutated IDH1 comprised more than 10% of the cell lysate, strong fluorescence was observed. However, when the proportion of mutated cells was below 5%, fluorescence was difficult to distinguish from the background.

Next, in order to determine the detection limit of the ratio of mutated IDH1/wild-type IDH1, we mixed 3.0 mg ml^–1^ cell lysate from mutant cells with the same concentration of wild-type cell lysate in various ratios. When mutated IDH1 comprised more than 10% of the cell lysate, strong fluorescence was observed. However, when the mutated cells accounted for less than 5% of the solution, fluorescence was difficult to distinguish from background intensity (Figure 3). The results indicate that the device can detect the *IDH1-R132H* mutation if the sample contains more than 10% of mutated IDH1. However, because the data shown here were determined using artificial cell lines overexpressing mutated or wild-type IDH1, further studies are needed to explore the detection threshold in clinical samples. 

### 3.2. Patient characteristics and protein extraction from tumor samples

Next, we analyzed brain tumor samples to investigate the utility of our method in the clinical setting. We analyzed tumors from 10 patients (mean age, 47 ± 20, three males and seven females); three GBMs, four LGGs, one gliomatosis cerebri, and two non-gliomas (Table 1). The DNA sequencing showed that all four LGGs harbored the *IDH1*-*R132H* mutation. We lysed 5–14 mg tumor samples (mean weight: 9.6 mg) in 200 μl RIPA buffer. The lysates contained 1.8–3.5 (mean, 2.9 ± 0.5) mg ml^–1^ of protein. 

**Table 1.** Patient characteristics and summarized results.

Case no.	Age	Sex	Pathological diagnosis	Sample weight (mg)	Protein lysate concentration (mg ml^–1^)	Amount of total protein (μg)	DNA sequencing	Immuno-wall	Mutant allele frequencies (%)
1	30	F	Anaplastic astrocytoma (grade III glioma)	11	2.71	542.4	Mut	Mut	31.5
2	53	M	Gliomatosis cerebri (grade III glioma)	7	2.89	578.4	WT	WT	0
3	50	F	Diffuse astrocytoma (grade II glioma)	10	3.47	694	Mut	Mut	45.8
4	78	F	Glioblastoma (grade IV glioma)	11	2.69	538	WT	WT	0
5	19	F	Dysembryoplastic neuroepithelial tumor	9	3.21	642.2	WT	WT	0
			(DNT, non-glioma)						
6	58	F	Pineal parenchyma tumor	5	1.86	371.6	WT	WT	0
			of intermediate differentiation (PPTID, non-glioma)						
7	59	M	Glioblastoma (grade IV glioma)	10	2.56	511.4	WT	WT	0
8	25	F	Anaplastic astrocytoma (grade III glioma)	12	2.89	578.6	Mut	Mut	34.7
9	47	M	Glioblastoma (grade IV glioma)	14	3.56	712.8	WT	WT	0
10	48	M	Diffuse astrocytoma (grade II glioma)	7	2.35	470.2	Mut	Mut	27.1

### 3.3. Immuno-wall assay of tumor samples

**Figure 4.**
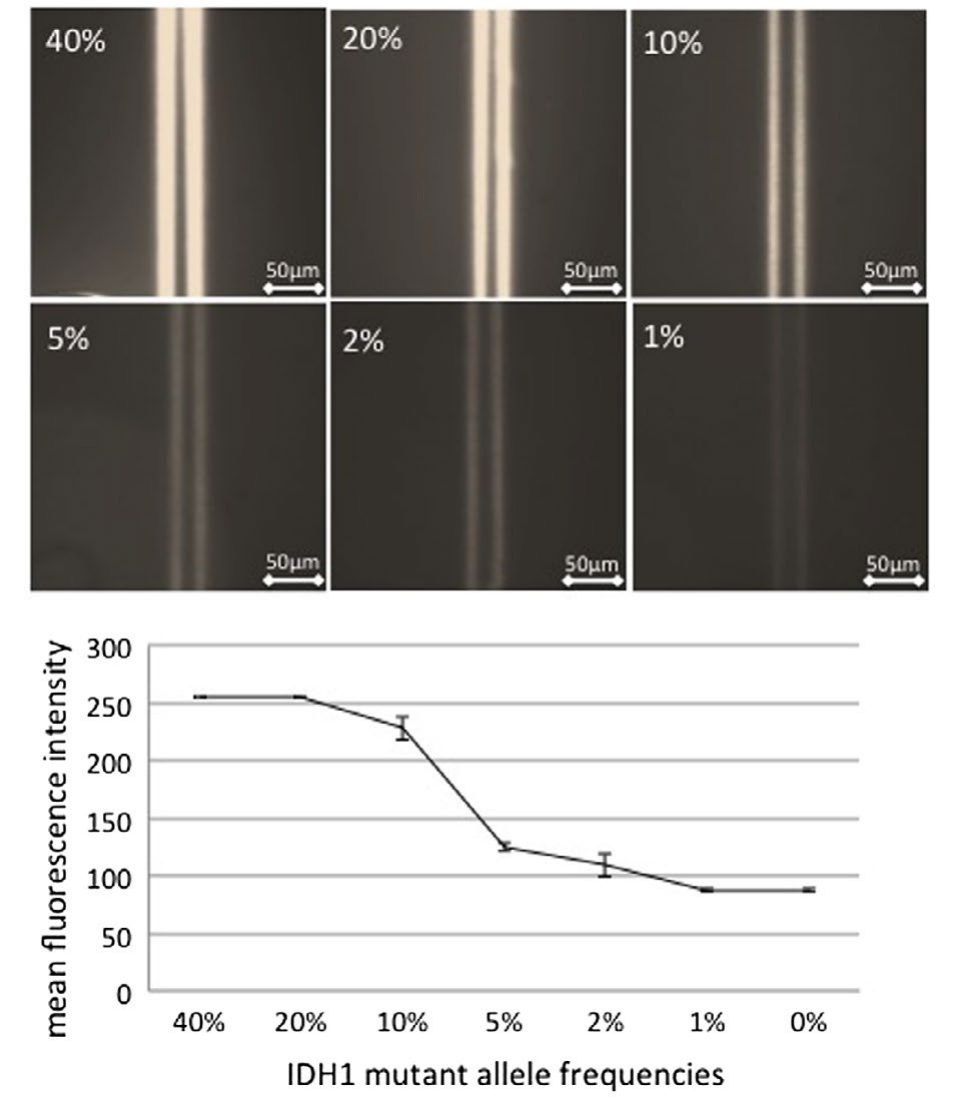
Effect of the mutant allele frequency in clinical samples on the detection of mutated IDH1. The lysate from a tumor sample with a mutant allele frequency of 40% was mixed with the lysate from a tumor sample with wild-type IDH1 such that the mutant allele frequency decreased stepwise from 40% to 20, 10, 5, 2, and 1%. The immuno-wall assay IDH1-R132H detected the mutant allele frequency in samples containing as low as 10% mutant IDH1.

All four LGGs containing the *IDH1*-*R132H* mutation as determined by DNA sequencing displayed positive intensity in the immuno-wall assay. Next, we evaluated the mutant allele frequencies (MAFs) by pyrosequencing the LGG samples exhibiting the *IDH1*-*R132H* mutation (Table 1). A tumor sample with an MAF of 40% was selected, and its lysate was mixed with another lysate from a tumor with wild-type IDH1 such that the MAF was decreased stepwise from 40% to 20, 10, 5, 2, and 1%. Our immuno-wall assay for *IDH1-R132H* detected as low as 10% of MAF (Figure 4). The detection limit in clinical samples was consistent with that in cell lines.

### 3.4. Detection of tumor border with immuno-wall assay

**Figure 5.**
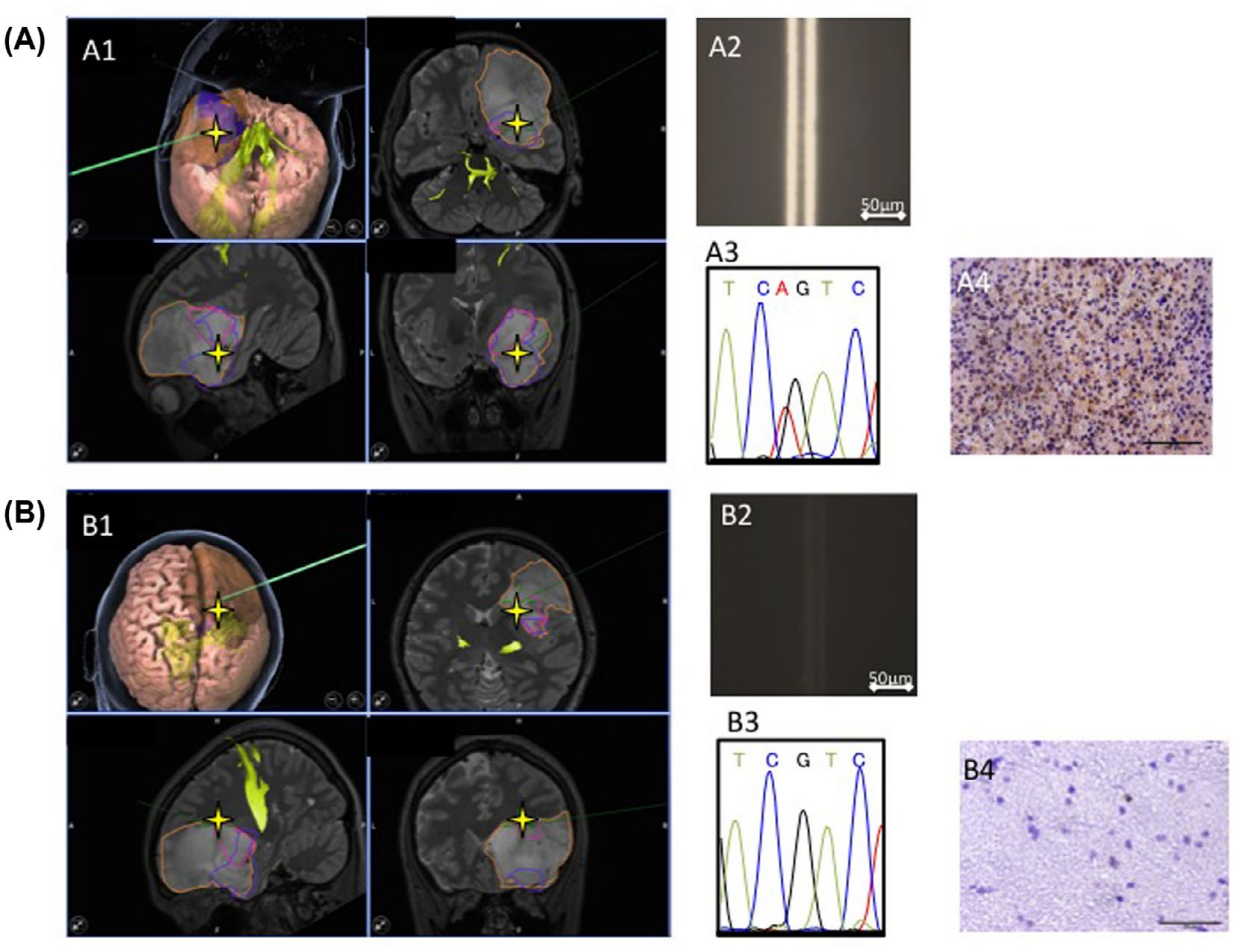
Tumor boundary detected using the immuno-wall assay. During tumor removal surgery in a patient, two specimens were collected. (A1, A2) A specimen from the center of the tumor tested positive in the immuno-wall assay. (B1, B2) In contrast, a specimen from the margins of the tumor, which appeared normal, tested negative. (A3, A4, B3, B4) The *IDH1* status of these samples was confirmed using immunohistochemistry and DNA sequencing.

As the immuno-wall was able to detect mutated cells with an MAF as low as 10%, we sought to utilize the device to estimate the gross tumor margin. A representative case of diffuse astrocytoma (Patient #8 in Table 1) is shown in Figure 5. During tumor removal surgery for this patient, two specimens were collected. A specimen from the center of the tumor showed positive intensity in the immuno-wall (Figure 5(A)), while, a specimen from the marginal region that appeared normal was negative (Figure 5(B)). The *IDH1* status of these samples agreed with the immunohistochemistry and DNA sequencing results. These data demonstrate that our immuno-wall assay can guide surgeons to the boundary between the tumor and surrounding normal brain. The immuno-wall assay requires only 15 min, indicating that the assay can be used as an intraoperative modality to image the border between the tumor and normal brain. 

## 4. Discussion

In this study, we established a highly sensitive antibody-based device to detect the *IDH1-R132H* mutation in glioma. Several techniques for detecting the *IDH1* mutation were recently developed, such as modified PCR protocols.[20 ^[21]^ ^[22]^–23] Even the most rapid method requires more than 60 min to obtain results. Our immuno-wall device detected the *IDH1* mutation within 15 min with substantially high sensitivity. Our immuno-wall generates an immunoreaction in microchannels fabricated using a photo-polymerizing polymer. This device enables the analysis of the *IDH1* status using a small sample, which shows potential for intra-operative rapid diagnosis.

Our immuno-wall device was constructed using a high concentration of streptavidin (10 mg ml^–1^) mixed with photoreactive polymer. Then primary antibody was immobilized to both sides of the walls via biotin–avidin binding. We estimated that the final density per area of primary antibody at the surface of the immuno-wall was approximately 0.155 μg mm^–2^ because the primary antibody was concentrated in the reaction with a high concentration of streptavidin during the 1-h incubation. We estimated the density of primary antibody at the bottom of the plate by using sandwich enzyme linked immunosorbent assay (ELISA; this method has high specificity) by using two antibodies and found a value of approximately 0.0014–0.0057 μg mm^–2^. Furthermore, fluorescence of the immuno-wall was integrated because the fluorescence signal extended laterally and all extended signals were observed from above. In contrast, the Western blotting signals were not integrated spatially because the membrane transfer signal was observed through a monolayer. A high density of primary antibody and integrated fluorescence increase the sensitivity of the immuno-assay compared to other immunoassays such as ELISA and Western blotting. Additionally, molecule movement is limited in the microchannels, enabling rapid detection because the primary antibody can capture molecules very quickly.

The rapid detection of *IDH1* mutation may also assist in determining the extent of tumor removal. During a surgical resection of glioma, it is difficult to visually differentiate the tumor from the surrounding normal tissue.[24] Intra-operative *IDH1* profiling may be useful for detecting the border between the tumor and normal brain. In this study, we evaluated a representative LGG case in which IDH1-R132H was detected at the center of the tumor concurrent with wild-type IDH1 at the margin. 

The emergence of molecular-targeting anti-cancer agents requires an individual molecular profiling of tumors performed in a non-invasive manner. ‘Precision medicine’ for cancer patients trends toward sensitive analysis of circulating tumor cells or cell-free DNA in liquids such as blood and cerebrospinal fluid.[25,26] In this regard, the rapid molecular diagnostic method developed in this study may be an important milestone in precision medicine. In this study, we demonstrated that our immuno-wall assay could detect mutated IDH1 in a tissue lysate containing 10% *IDH1* mutant allele at a protein concentration of 1.0–1.5 mg ml^–1^ (Figure S2). Because the minimal sample volume for a microchannel is 0.33 μl, 500 ng protein or 500 cells is sufficient for determining *IDH1* status. Total protein concentration in cerebrospinal fluid is more than 1.0 mg ml^–1^ in patients with glioma.[27] Recent studies reported that the number of circulating tumor cells in the blood of glioma patients is approximately 10 cells ml^–1^.[28,29]. These suggest that there is a possibility that the *IDH1* status can be determined with our device using cerebrospinal fluid and blood.

Some challenges remain before this diagnostic tool can be applied clinically, but our immuno-wall assay may lead to rapid and highly sensitive detection of the *IDH1-R132H* mutation preoperatively and intra-operatively. Clinical application, using a newly developed portable CCD camera, is currently under development; this will facilitate rapid on-site diagnosis. Our immuno-wall device will enable non-invasive rapid liquid biopsy in precision medicine.

## Disclosure statement

No potential conflict of interest was reported by the authors.

## Funding

This work was supported by a JSPS KAKENHI [grant number 25462242]; Grant-in Aid for Scientific Research on Innovative Areas from the Ministry of Education, Culture, Sports, Science and Technology (MEXT) of Japan [grant number 23107010]; Practical Research for Innovation Cancer Control from Japan Agency for Medical Research and development, AMED; by ‘Knowledge Hub Aichi’, Priority Research Project from Aichi Prefectural Government; by the Platform for Drug Discovery, Informatics, and Structural Life Science (PDIS) from AMED; and by the Regional Innovation Strategy Support Program from MEXT of Japan.

## Supplemental data

Supplemental data for this article can be accessed here. [http://dx.doi.org/10.1080/14686996.2016.1227222]

## Supplementary Material

TSTA_1227222_suppl.zip
