# Supplementary figures and images for: An immuno-wall microdevice exhibits rapid and sensitive detection of IDH1-R132H mutation specific to grade II and III gliomas
Source: Sci Technol Adv Mater. 2016 Oct 4;17(1):618–25. doi: 10.1080/14686996.2016.1227222 (PMC5101859; doi:10.1080/14686996.2016.1227222)

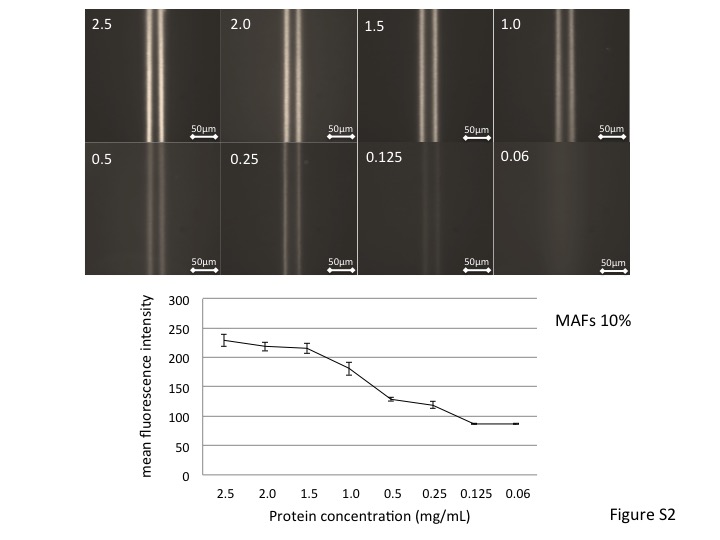

Supplement: TSTA_1227222_suppl.zip [file tsta_a_1227222_sm9283.zip › FigS2.jpg]

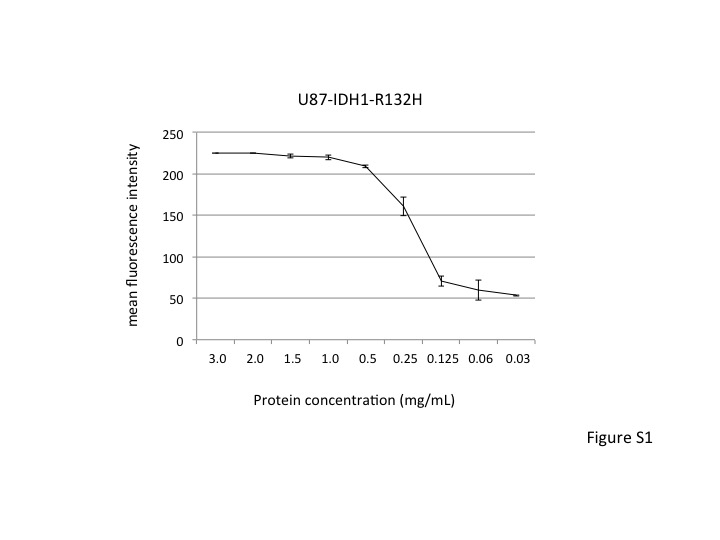

Supplement: TSTA_1227222_suppl.zip [file tsta_a_1227222_sm9283.zip › FigS1.jpg]
